# Association between Body Mass Index and Bone Mineral Density in Patients Referred for Dual-Energy X-Ray Absorptiometry Scan in Ajman, UAE

**DOI:** 10.4061/2011/876309

**Published:** 2011-05-22

**Authors:** Tarek Fawzy, Jayakumary Muttappallymyalil, Jayadevan Sreedharan, Amal Ahmed, Salma Obaid Saeed Alshamsi, Mariyam Saif Salim Humaid Bin Bader Al Ali, Khawla Ahmed Al Balsooshi

**Affiliations:** ^1^Department of Radiodiagnosis, Gulf Medical College Hospital and Research Centre, P.O. Box 4184, Ajman, UAE; ^2^Research Division, Gulf Medical University, P.O. Box 4184, Ajman, UAE

## Abstract

Body Mass Index (BMI) is a good indicator for measurements of Bone Mineral Density (BMD) which measures the density of minerals present in the bones using a special scan. This study was conducted to assess the association between BMI and status of BMD among 101 individuals who underwent Dual-Energy X-ray Absorptiometry (DEXA) scan. 39 subjects had normal and 62 had low bone mineral density. BMD was low in 82.4% of people with normal BMI, 78.1% among overweight, and 44.2% among obese. There was a statistically significant association between these two variables (*P* < .001). Low BMD was recorded in 59.1% of females and 76.9% of males. Association between advancing age and lower BMI is an important risk factor in the occurrence of low BMD.

## 1. Introduction

Bone mineral density (BMD) test measures the density of minerals present in the bones using a special scan. This can be used to assess the strength of bones. Bones naturally become thinner as you grow older because existing bone is broken down faster than the new bone made. As a result of this, calcium and other minerals decrease in the bones and they become light in weight, less dense, and more fragile. The bones might break if it goes thinner and weaker. Therefore, thicker bones take longer time to get osteoporosis. Although osteoporosis can occur in men, it is most common in women older than 65 years of age. Prospective studies by Ravn et al. [1] and Bjarnason and Christiansen [2] indicates that early postmenopausal women who have low BMI lose more bone compared to those with higher BMI tertiles. Studies conducted by Van der Voort et al. [3, 4] illustrates that thinness is related to both osteoporosis and increased fracture risk; hence, low BMI was included in the risk assessment tools for evaluation of osteoporosis and osteoporotic fracture risk as suggested by Eddy et al. [5] and National Osteoporosis Foundation [6] and Black et al. [7]. Studies from different authors emphasize the importance of exercise on BMD. Chan et al. [8] and Loud et al. [9] in their study indicate that exercise at a younger age helps to maximize the BMD. Family history of osteoporosis and fracture, tobacco consumption, and dietary habits are some of the factors related to the occurrence of osteoporosis. Among the anthropometric variables, low BMI and low weight are also interrelated with the occurrence of osteoporosis. A study conducted by Iqbal et al. [10] found that low BMI is a good indicator for referral of women less than 60 yr for measurements of BMD. 

The present study was conducted to assess the association between BMI and status of BMD among individuals referred to Department of Radio diagnosis for DEXA scan.

## 2. Materials and Methods

The present retrospective study was conducted in Gulf Medical University, Ajman, UAE. For the study, people who were referred to the Department of Radio diagnosis for DEXA scan were selected. People who underwent DEXA scan from January till September 2009 formed the study subjects. Those patients referred for DEXA scan had metabolic syndromes, bone diseases, and few had difficulty in walking. As it is a case record analysis “agree to participate” does not arise. DEXA Scanning was performed by two technicians, and the interpretation was done by one radiologist. For the present study, 25- to 80-year old individuals of both genders who attended various departments of Gulf Medical College Hospital and Research Centre, Ajman, and whose densitometries recovered were included. The tool used for data collection consisted of sociodemographic details like age and gender. Height and weight were measured and respondents were dressed in light clothes and did not wear shoes. BMI was calculated from the height and weight recorded while performing DEXA scan. The BMI was calculated based on the formula weight (kg)/[height (m)]^2^. The standard categorisation of BMI by CDC [11] indicates less than 18.5 as Underweight, 18.5–24.9 as Normal, 25.0–29.9 as overweight, and 30.0 and above as obese.

Dual-Energy X-ray Absorptiometry (DEXA) is the most accurate way to measure BMD. A certified technician measured BMD at the femoral neck and the lumbar spine (L2 to L4) using a DEXA densitometer. WHO criteria were used for categorizing the respondents based on DEXA results. The statistical analysis included descriptive statistics to rule out the association between sociodemographic characteristics and DEXA scan result, BMI, and status of BMD. The statistical tests used in this study were chi-square test and logistic regression. The variables considered were gender, age, and BMI. The difference between the subjects was considered significant if the *P* value was less than  .05.

## 3. Results

The study population consisted of 101 individuals. Distribution of gender showed that 87.1% were females and the remaining were males. The sex ratio was found to be male : female = 1 : 6.7. Age ranged from 25 to 80 years. The mean and standard deviation of age was 50.5 ± 11.5 years. The respondents were classified in to three broad age groups, 25–39 years, 40–59 years, and 60 years and above. About 68% in the study were in the age group of 40–59 years. More than 50% of the subjects were obese. A detailed description is given in Table 1.

The results of the DEXA scan of 101 respondents show that 35.6% were osteopaenic and 25.7% had osteoporosis. 38.6% had normal DEXA scan results. Among patients with osteopaenia and osteoporosis, about 77.4% were overweight or obese. In the present study, none of the participants in the age group 25–39 years had osteoporosis. Maximum number of osteoporosis cases was seen in the age group of 60 years and above. In the age group of 40–59 years, only 23% had osteoporosis. The details of DEXA scan results are given in Table 2.

For further analysis, the whole group based on the DEXA scan result was divided as normal and low bone mineral density. There were 39 subjects with normal BMD and 62 with low BMD. BMD was low in 82.4% of people with normal BMI, 78.1% among overweight and 44.2% among obese. The association between BMI and BMD was found to be statistically significant (*P* < .001). Agewise variations showed that 33.3% of those in age group 25–39 years, 60.9% in 40–59 years of age, and 88.2% of those aged 60 years and above had low BMD. There was a statistically significant association between BMD and age (*P* < .01). The details are depicted in Table 3.

In the present study, age and BMI were the two factors found to be statistically associated with BMD. These two variables were put into a binary logistic regression model to find the crude and adjusted Odds Ratio (OR). The results of this analysis showed that compared to normal BMI, the chances of getting low BMD in overweight persons are 50% less. This association was not statistically significant. But among obese persons, the chance of getting low bone mineral density was 89% less compared to normal, which was statistically significant. As far as age is concerned, compared to those in age group 25–39 years the chance of getting low bone mineral density in persons aged 40–59 years was 4.24 times higher, and this was statistically significant. But those with age 60 years and above, the chance of low bone mineral density is 23 times higher compared to those in the age group 25–39 years. This association was found to be statistically significant. The details are depicted in Table 4.

## 4. Discussion

Osteoporosis is a major public health problem all over the world. The epidemiology of osteoporosis is, however, not well known in most of the Middle East countries. The present study was conducted in Gulf Medical University, Ajman, UAE, to identify the factors associated with the occurrence of low BMD. The three factors considered included age, gender, and BMI. DEXA scan uses two different X-ray beams to assess the bone density in the spine and hip. Although osteoporosis involves the whole body, measurements of BMD at one site can be predictive of fractures at other sites. Less X-ray beams pass through strong bones. DEXA can measure as little as 2% of bone loss per year. It is quick, and very low doses of radiation are used, but it is more costly when compared to ultrasound testing.

The correlation between BMD at the femoral neck and BMI observed was highly positive in a crosssectional study conducted among postmenopausal women by Steinschneider et al. [12]. The findings suggest that the increased BMD commonly reported in overweight women may result from soft tissue interference with BMD determination by DEXA. A hospital-based study conducted in elderly males by Paniagua et al. [13] observed that 37.3% were normal, 35.6% were osteopaenic and 27.1% were osteoporotic. In the same study, 35.6% of patients had normal BMI, 3.4% were underweight, 47.5% overweight, and 13.6% obese. Overweight and obese men were more likely to have osteoporosis and osteopaenia. Similar studies by Felson et al. [14], Nguyen et al. [15] and Baheiraei et al. [16] also reported the consistent finding that lower BMI was associated with lower BMD. 

The previous literature of Baheiraei et al., [16] Jones et al. [17], and Nguyen et al. [18] indicated that advancing age was associated with low BMD. In this study also we observed an association between age and bone mineral density. The chance of low bone mineral density among people with age 60 and above is 23 times higher compared to those with age 25–39 years. 

This study is an attempt to address one of the important public health problems which can be controlled if preventive measures are taken at an early stage. More about the risk factors could not be investigated as this is a retrospective case record analysis.

## 5. Conclusion

The results of this study suggest that advancing age and lower BMI are important risk factors for the occurrence of low BMD. Further studies are required to investigate the effect of other factors like exposure to sunlight, calcium intake, and other habits like smoking, diet, and so forth.

## Figures and Tables

**Table 1:** Distribution of respondents according to age, gender, and BMI.

Variable	Group	No.	%
Gender	Male	13	12.9
	Female	88	87.1

Age	25–39	15	14.9
	40–59	69	68.3
	60 and above	17	16.8

BMI	Normal	17	16.8
	Overweight	32	31.7
	Obese	52	51.5

**Table 2:** Distribution of bone mineral density according to independent factors.

		Status of bone mineral density						
Independent factors		Normal		Osteopaenic		Osteoporosis		Total
		No.	%	No.	%	No.	%	
BMI	Normal	3	17.6	7	41.2	7	41.2	17
	Overweight	7	21.9	12	37.5	13	40.6	32
	Obese	29	55.8	17	32.7	6	11.5	52

Age (in Years)	25–39	10	66.7	5	33.3	—	—	15
	40–59	27	39.1	26	37.7	16	23.2	69
	60+	2	11.8	5	29.4	10	58.8	17

Total		39	38.6	36	35.6	26	25.7	101

**Table 3:** Association between bone mineral density and independent factors.

		Status of bone mineral density					*P*
Independent factors		Normal		Low BMD		Total	
		No.	%	No.	%		
BMI	Normal	3	17.6	14	82.4	17	*P* < .001
	Overweight	7	21.9	25	78.1	32	
	Obese	29	55.8	23	44.2	52	

Age (in Years)	25–39	10	66.7	5	33.3	15	*P* < .01
	40–59	27	39.1	42	60.9	69	
	60+	2	11.8	15	88.2	17	

Total		39	38.6	62	61.4	101	

**Table 4:** Crude and adjusted odds ratio of independent variables.

Independent factors		Crude		Adjusted	
		OR	CI	OR	CI
BMI	Normal	1	—	1	—
	Overweight	0.77	0.17–3.44	0.50	0.10–2.55
	Obese	0.17	0.04–0.66	0.11	0.02–0.50

Age (in Years)	25–39	1	—	1	—
	40–59	3.11	0.95–10.10	4.24	1.09–16.53
	60+	15.0	2.42–93.0	22.94	3.12–168.68
